# Advances in mitochondria-centered mechanism behind the roles of androgens and androgen receptor in the regulation of glucose and lipid metabolism

**DOI:** 10.3389/fendo.2023.1267170

**Published:** 2023-10-13

**Authors:** Lijun Yin, Shuo Qi, Zhiqiang Zhu

**Affiliations:** ^1^ School of Sport, Shenzhen University, Shenzhen, China; ^2^ School of Sport Health, Shandong Sport University, Jinan, China

**Keywords:** androgen, androgen receptor, mitochondria, glucose metabolism, lipid metabolism, signaling

## Abstract

An increasing number of studies have reported that androgens and androgen receptors (AR) play important roles in the regulation of glucose and lipid metabolism. Impaired glucose and lipid metabolism and the development of obesity-related diseases have been found in either hypogonadal men or male rodents with androgen deficiency. Exogenous androgens supplementation can effectively improve these disorders, but the mechanism by which androgens regulate glucose and lipid metabolism has not been fully elucidated. Mitochondria, as powerhouses within cells, are key organelles influencing glucose and lipid metabolism. Evidence from both pre-clinical and clinical studies has reported that the regulation of glucose and lipid metabolism by androgens/AR is strongly associated with the impact on the content and function of mitochondria, but few studies have systematically reported the regulatory effect and the molecular mechanism. In this paper, we review the effect of androgens/AR on mitochondrial content, morphology, quality control system, and function, with emphases on molecular mechanisms. Additionally, we discuss the sex-dimorphic effect of androgens on mitochondria. This paper provides a theoretical basis for shedding light on the influence and mechanism of androgens on glucose and lipid metabolism and highlights the mitochondria-based explanation for the sex-dimorphic effect of androgens on glucose and lipid metabolism.

## 1 Introduction

Androgens, as steroid sex hormones, primarily affect the growth and development of the male reproductive system. In recent years, many studies have revealed that androgens can also regulate glucose and lipid metabolism upon binding to androgen receptors (AR) (1, 2). Evidence from pre-clinical and clinical studies has reported that in male individuals, androgens promote energy expenditure, alleviate dyslipidemia, and prevent the occurrence and progression of obesity. Disorders of glucose and lipid metabolism in men are associated with age, obesity, and hypogonadism (decreased androgens and/or AR levels) (3) or in male animals with androgen deficiency (4, 5). Androgen levels decrease with worsening obesity, and excessive fat impedes the synthesis of androgens by inhibiting the “hypothalamic-pituitary-gonadal” axis in the central nervous system and enhancing the secretion of aromatase, in peripheral tissues such as the white adipose tissue, which promotes the conversion of androgens to estrogen (6), further reducing androgens levels and deteriorating metabolism.

In addition to the gonadal organs, AR are also expressed in many major metabolic tissues, including the skeletal muscle, liver, pancreas, and brain, suggesting their important involvement in metabolism. Previous evidence has demonstrated that the decrease in the expression and activity of AR is closely related to the disorders of glucose and lipid metabolism. The blood lipids (e.g., free fatty acid and triglyceride) and cytokines (e.g., leptin) in mice with global AR knockout (ARKO) or tissue-specific knockout (e.g., liver and skeletal muscle) are significantly increased, and they are prone to suffer from obesity, insulin resistance (IR), and liver fibrosis (7, 8). Although multiple studies have reported the important role of androgens in the regulation of glucose and lipid metabolism, the underlying mechanisms have not been fully elucidated. An increasing body of evidence has revealed that patients with obesity, diabetes, and hypogonadism often have mitochondrial dysfunction (9, 10), and supplementation with exogenous testosterone can improve both mitochondrial function and metabolism (11), suggesting that the regulation of mitochondrial function by androgens may be one of the important ways to affect glucose and lipid metabolism. However, the specific mechanism behind this has not been systematically reported.

As the metabolic center of energy substrates, mitochondria produce a large amount of adenosine triphosphate (ATP) to meet the energy needs of cells. In addition, mitochondria also play an important role in influencing intracellular calcium levels, reactive oxygen species (ROS) production, regulating innate immune responses, and apoptosis (12). Mitochondrial dysfunction is strongly related to the disorders of glucose and lipid metabolism, which manifests in various ways. Firstly, impaired mitochondria can hinder glucose uptake by inhibiting the activity of adenosine monophosphate (AMP)-activated protein kinase (AMPK), an important “energy sensor,” as well as glucose transport protein 4 (GLUT4) (13). Secondly, dysfunctional mitochondria can increase the activity of mTOR/S6K and JNK, leading to the serine phosphorylation of insulin receptor substrate proteins (14). This phosphorylation interferes with the normal insulin signaling cascade, impairing glucose and lipid metabolism regulation. Lastly, mitochondrial dysfunction may lead to adipocyte dysfunction, resulting in the release of excessive circulating free fatty acids and the ectopic deposition of fat (15). This abnormal fat distribution can lead to lipotoxicity, further exacerbating glucose and lipid metabolism disorders. Thus, maintaining the density, quality, and function of mitochondria under optimal conditions is essential for glucose and lipid metabolism.

Androgens can regulate glucose and lipid metabolism by affecting mitochondrial density, quality, and function. In hypogonadal men with diabetes or in male rodents with androgen deficiency, decreased mitochondrial density, abnormal mitochondrial morphology, and lower ATP production were observed, while exogenous testosterone supplementation effectively alleviated mitochondrial dysfunction and improved glucose and lipid metabolism (16–18). Although many studies, including a recent published review article summarized the effects of androgens on mitochondrial structure, function, and ROS production (19), the specific underlying molecular mechanism has not been systematically revealed, and the discussion about the mitochondria-based mechanism behind the effects of androgens on glucose and lipid metabolism needs further demonstration. In this study, we analyzed the effect of androgens and AR on mitochondrial density, quality control, and function in males and females, with a particular emphasis on the advances in mitochondria-based molecular mechanisms of androgens in regulating glucose and lipid metabolism. This study provides a theoretical basis for further understanding the sex-dimorphic effect and mechanism of androgens/AR signaling in the regulation of glucose and lipid metabolism.

## 2 The sex-dimorphic regulatory impact of androgens and AR on glucose and lipid metabolism

### 2.1 Inhibited androgens/AR negatively affects glucose and lipid metabolism in males

The most well-known androgens include testosterone, androstenedione, dehydroepiandrosterone (DHEA), dehydroepiandrosterone sulfate (DHEA-S), and dihydrotestosterone (DHT). Androstenedione, DHEA, and DHEA-S are produced mainly by the adrenal cortex, and in relatively small quantities, while testosterone is mostly secreted by Leydig cells (20). DHEA and DHEA-S can be converted to testosterone in other tissues, and a small quantity of testosterone can also be converted to DHT with the help of 5-alpha reductase. Unlike testosterone, DHT does not play a notable role in maintaining male physiology in adulthood; it is mainly involved in prostate enlargement and male-pattern hair loss in adulthood (21). Thus, testosterone is the predominant and most active androgen.

As previously mentioned, androgens play a vital role in the regulation of glucose and lipid metabolism in patients with chronic diseases such as obesity and diabetes, which is mainly achieved by binding to AR (22, 23). AR belong to the nuclear receptor superfamily with high expressions not only in gonadal organs, but also in multiple major metabolic tissues including the brain, skeletal muscle, adipose tissue, liver, and pancreatic islets (24, 25). AR bind to heat shock proteins in an inactivated state and then undergo heterodimerization upon androgen activation. After that, AR translocate into the nucleus, bind to androgen response elements (AREs) in the gene sequence of target molecules, and regulate the expression of target genes with the coordination of co-regulatory factors (26).

Inhibition of androgens and/or AR is strongly associated with disorders of glucose and lipid metabolism. Low serum testosterone levels can be used as a risk predictor of diabetes in men (27). Evidence from clinical studies has suggested that hyperglycemia, IR, hypertension, and lipid metabolism disorders are frequently observed in hypogonadal men, with more severe disorders in patients with lower androgen levels (27, 28). Exogenous testosterone supplementation is effective in alleviating or even reversing these disorders (29–32). In addition, pre-clinical studies also demonstrated that adverse changes in blood lipids (e.g., increase in TG, LDL levels, and decrease in HDL) and the development of IR were observed in male mice with castration (16) or testicular feminization (33). Knockdown of hypothalamic AR is closely associated with reduced insulin sensitivity and central obesity (34). In peripheral tissues, androgens/AR signaling regulates glucose and lipid metabolism by affecting lipolysis, glucose uptake, and pancreatic function (35, 36). AR antagonists markedly decreased the energy consumption efficiency and lipid metabolism in the skeletal muscle of mice (37). Further, adipose-specific AR gene knockout mice were more vulnerable to high-fat diet-induced central obesity and glucose intolerance (35). Interestingly, testosterone supplementation and/or selective androgen receptor modulators (e.g., ostarine) were reported to improve these disorders in glucose and lipid metabolism, along with the reduction of leptin and adiponectin levels (38).

### 2.2 Excessive androgens and overactivation of AR hinder glucose and lipid metabolism in females

The role of androgens in glucose and lipid metabolism in females remains different. Excessive testosterone (hyperandrogenemia) is the main condition in females with polycystic ovary syndrome (PCOS) and a risk factor for the development of IR and type 2 diabetes (39). Recent studies have reported that hyperandrogenemia induces disorders of glucose and lipid metabolism at the central and peripheral levels, promoting the development and progression of obesity and type 2 diabetes in both female individuals and rodents with PCOS (40, 41).

At the central level, testosterone alters the function of kisspeptin and gonadotropin-releasing hormone (GnRH) neurons in the hypothalamic arcuate nucleus of female mice at premature and adult stages in an AR-dependent manner. Additionally, it negatively affects the activity of energy metabolism-related neurons in the hypothalamic arcuate nucleus (e.g., increases appetite and energy intake by elevating spiny mouse state-related protein: agouti related protein (AgRP) neurons and decreasing levels of allantoinogen proopiomelanocortin (POMC) neurons). Excessive androgens prompt obesity by reducing the communication between the hypothalamic nucleus and brown adipose tissue, and decreasing the function of leptin. In addition, AgRP co-localizes with AR in the hypothalamus, androgens regulate metabolic homeostasis by affecting these neurons, and prenatal testosterone or DHT exposure decreases the co-localization of AgRP and insulin receptors, which subsequently affects hepatic insulin sensitivity (42–44). Selective depletion of AR in the mediobasal hypothalamus (MBH) neurons protected against high-fat diet- and DHT-induced hyperinsulinemia and IR in female mice (45).

At the peripheral level, excessive androgens negatively regulate metabolism in multiple metabolic tissues including the adipose tissue, liver, pancreas, and skeletal muscle. Excessive androgens, on the one hand, induce disorders of lipid metabolism by promoting visceral fat deposition, enlarging adipocytes, enhancing inflammatory response, macrophage infiltration, and apoptosis in women (39). On the other hand, excessive DHEA contributes to the disorder of glucose metabolism through deteriorating oxidative damage, pancreatic β-cell dysfunction, and dysbiosis of intestinal ecology (46), which eventually triggers the development of diabetes (45). Moreover, this strong connection between excessive androgens and adverse changes in metabolism exists in females, even during childhood and adolescence (47), and hyperandrogenemia in early adulthood is an independent risk factor for abnormal glucose metabolism in middle age (48).

**Figure 1:**
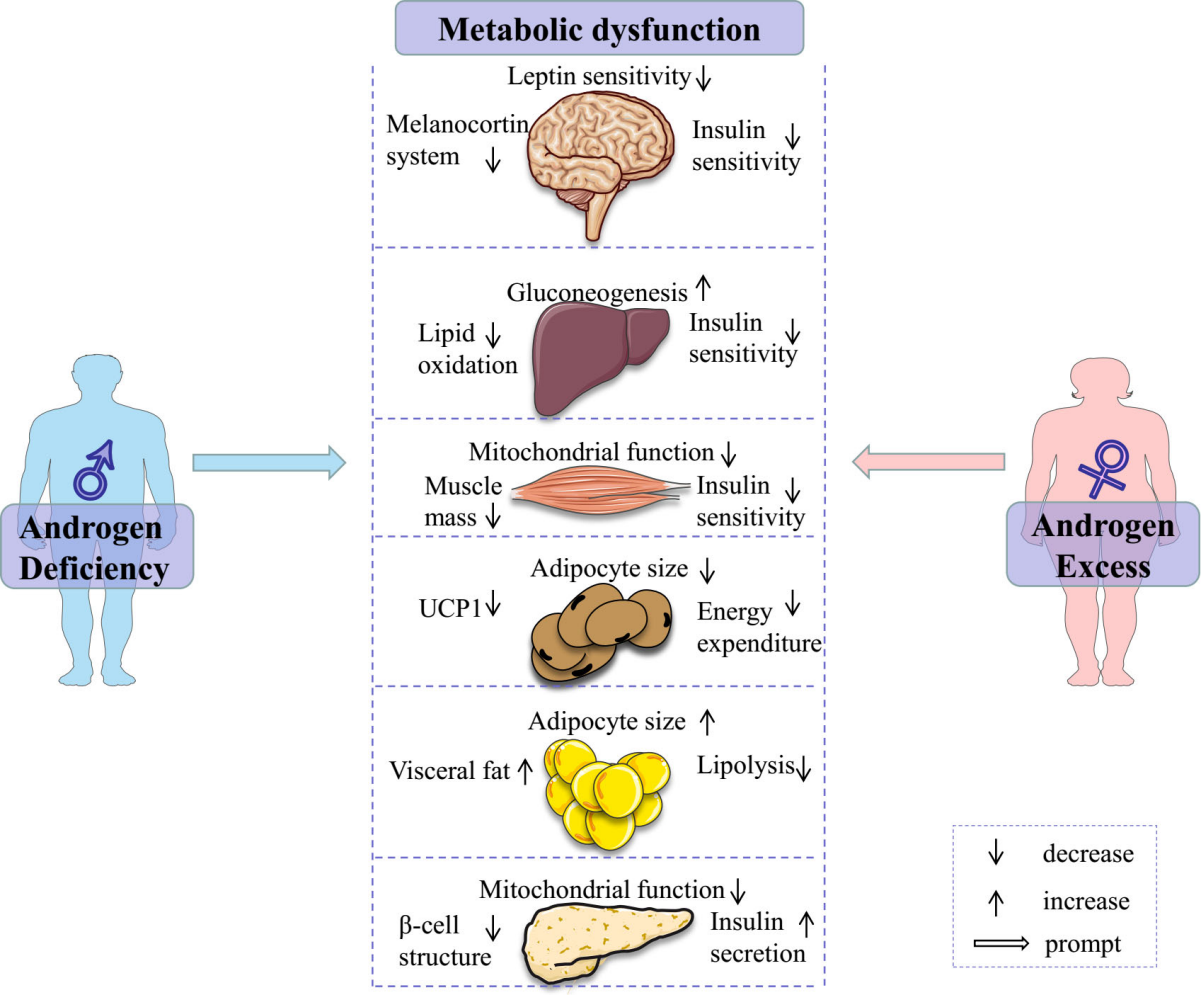
Androgen deficiency in males and androgen excess in females contribute to metabolic dysfunction through various ways. Some elements of this figure are from Servier Medical Art, https://smart.servier.com.

In summary, evidence from pre-clinical and clinical studies reveal the consistent regulatory roles of androgens/AR in glucose and lipid metabolism, with clear sex-dependent differences (**Figure 1**). In males, the decrease in androgens/AR signaling contributes to the disorder of glucose and lipid metabolism and is considered a promising predictor of chronic diseases. In females, excessive androgen is strongly associated with adverse glucose and lipid metabolism. These evidences suggest that serum androgens and AR protein levels in metabolic tissues are non-negligible factors in deciphering the mechanism behind sexual dimorphism in metabolic health, and in establishing a precise sex-based therapeutic strategy for the prevention and treatment of metabolic diseases.

## 3 Mitochondrial dysfunction is strongly associated with impaired glucose and lipid metabolism in obesity and diabetes

Prolonged overload by nutrient substrates causes mitochondrial dysfunction (49), which can occur in multiple ways, including mitochondrial swelling, smaller mitochondrial cristae, imbalanced mitochondrial dynamics, and abnormalities in the mitochondrial respiratory chain (50, 51). Mitochondrial dysfunction is closely associated with abnormal energy metabolism, inflammatory response, IR (50, 52), and the occurrence of obesity, diabetes, and chronic renal disease (53–55). Clinical studies have reported that the damaged structure and dysfunction of mitochondria (56) including inefficient ATP production, excessive ROS (57–60), and decreases in mitochondrial biogenesis and activity of ATP synthase (61, 62) were clearly observed in patients with obesity or diabetes. In addition, the oxidative damage induced by excessive ROS and decreases in antioxidants, such as superoxide dismutase and glutathione peroxidase in diabetes also causes mitochondrial dysfunction (63). In turn, dysfunctional mitochondria are commonly associated with metabolic dysfunction and disease (64). In terms of lipid metabolism, dysfunctional mitochondria disrupt the balance between lipid usage and storage within cells. This is supported by research showing that inhibiting mitochondrial fission through decreasing *Drp1* leads to an increase in fatty acid content in lipid droplets and a decrease in fatty acid oxidation (65). Additionally, dysfunctional mitochondria contribute to the accumulation of bioactive lipid intermediates, such as diacylglycerols and ceramides, in the liver and skeletal muscle. This accumulation is caused by decreased substrate oxidation levels, which further exacerbates diabetes by inhibiting insulin signaling (50). Regarding glucose metabolism, dysfunctional mitochondria have negative effects in various metabolic organs. At the central level, the impaired mitochondria reduce the glucose uptake and ATP production in the brain, which subsequently hinders the energy balance. This was supported by the evidence that the genetic removal of *Mfn2* in POMC neurons results in severe obesity characterized by excessive eating and reduced energy expenditure (66). At the peripheral level, the deletion of *Opa1* leads to the death of pancreatic β cells, impairing insulin secretion and systemic glucose regulation (67). In the liver, the absence of *Mfn2* causes mitochondrial fragmentation and triggers ER stress, leading to increased hepatic glucose production and impaired insulin signaling (68). Therefore, a vicious circle is formed between mitochondrial dysfunction and adverse glucose and lipid metabolism.

Many studies have indicated that mitochondria are potential targets for the prevention and treatment of obesity or type 2 diabetes (61). It has been reported that exercise or restriction of energy intake enhances skeletal muscle mitochondrial levels and oxidase activity, reduces body weight, and improves insulin sensitivity in patients with obesity and diabetes (50). In addition, a decrease in androgens/AR level in males with obesity and diabetes is often accompanied by mitochondrial dysfunction, as evidenced by a study showing that male rats experienced myocardial mitochondrial ROS accumulation and enhanced apoptosis after four weeks of castration, whereas these disorders did not occur until week eight in rats of the sham group (69). In addition, exogenous androgens supplementation improves mitochondrial function and glucose and lipid metabolism (70). Therefore, improving androgens/AR signaling may be a promising strategy to enhance mitochondrial function and improve glucose and lipid metabolism in males.

## 4 The mechanisms behind the regulatory role of androgens/AR in mitochondrial density, quality control, and function in males

Mitochondrial density and quality matter mostly for the normal function of mitochondria. Mitochondrial biogenesis is an important process for maintaining mitochondrial density, and decreased biogenesis can induce insufficient mitochondrial ATP production. The mitochondrial quality control system includes mitochondrial dynamics and mitophagy. Additionally, the integrity and function of the electron transfer chain (ETC) are vital for normal mitochondrial function. Androgens and androgen receptors can affect mitochondrial function through these aspects.

### 4.1 Androgens/AR regulate mitochondrial biogenesis

Mitochondrial biogenesis maintains mitochondrial quantity by bringing together materials such as lipids, proteins, and mitochondrial deoxyribonucleic acid (DNA) into existing mitochondria. There are approximately 1200 proteins in the mitochondria, 13 of which are encoded by mitochondrial genomic DNA (mtDNA), while the rest are encoded by nuclear DNA. Mitochondrial biogenesis requires a fine tune between the mitochondrial and nuclear genomes through “bidirectional regulation”. In anterograde regulation, proteins encoded by the nuclear genome are transferred to the mitochondria to perform functions (71), while in retrograde regulation, the signal generated from mitochondria passes into the nucleus to induce gene expression, which occurs when mitochondrial homeostasis is interrupted by hypoxia or mutation in mtDNA, or mitochondrial Ca^2+^ release (72, 73).

Mitochondrial biogenesis is mainly controlled by a network of transcription factors in the nucleus, including peroxisome proliferator-activated receptor γ (PPARγ), peroxisome proliferator-activated receptor α (PPARα), estrogen-related receptors (ERRs), cylic AMP (cAMP) response element-binding protein (CREB), forkhead box (FOXO), and peroxisome proliferator-activated receptor γ coactivator-1 (PGC-1α) (74–77). PGC-1α initiates mitochondrial biogenesis as a transcriptional coactivator after translocation from the cytoplasm to the nucleus upon phosphorylation by AMPK, and binds to transcription factors including PPARγ, PPARα, and CREB (75, 76). PGC-1α stimulates the expression of mitochondria-related genes, such as nuclear respiratory factors 1/2 (NRF-1 and NRF-2), transcription factors encoded by the nuclear gene that regulate cellular energy metabolism in the nucleus; influences mtDNA transcription and replication; and promotes mitochondrial function through the activation of mitochondrial DNA transcription factor A (TFAM) (78). TFAM is a key regulator of the mitochondrial levels and functions that directly regulate transcription, translation, and reconstruction of mtDNA within mitochondria (79, 80). Many studies have reported that the downregulation of PGC-1α was associated with abnormal mitochondrial biogenesis and diminished ATP production capacity, and the reduced mitochondrial biogenesis in hepatocytes by silica nanoparticles was also associated with the inhibition of the PGC-1α/NRF-1/TFAM pathway (81).

Testosterone promotes biogenesis by activating the AR/PGC-1α/TFAM pathway in the skeletal muscle (82). Evidence from pre-clinical studies showed that castration notably inhibits protein levels of PGC-1α and TFAM, and reduces mitochondrial biogenesis in the skeletal muscle of rats, mice, and pigs (82, 83). Exogenous testosterone supplementation upregulates the expression and activity of PGC-1α protein and enhances skeletal muscle mitochondrial biogenesis and energy expenditure, which eventually alleviates castration-induced mitochondrial dysfunction (17, 83). mRNA levels of genes involved in mitochondrial biogenesis were down-regulated in AR deficient mice (83). Evidence from cultured cells showed that testosterone induced the levels of *Pgc-1α* and *Tfam* genes in C_2_C_12_ myogenic cells (83) and increased the number of myotubular and mitochondrial DNA copies in myogenic cells, which was notably attenuated by an inhibitor of AR (84). Furthermore, recent evidence suggests that *Pgc-1α* and *Nrf-1* are directly regulated by AR. This is supported by the observation that these genes were found to be down-regulated in AR-deficient myofibers (85). Additionally, luciferin assay experiments have confirmed that AR promotes the expression of TFAM by binding to potential AR elements in the TFAM promoter (82).

Androgens/AR can also affect mitochondrial levels by regulating mtDNA. Mitochondria have their own genome that encodes important component proteins of the mitochondrial respiratory chain, including the seven subunits of complex I, cytochrome B (cytB) of complex III, and cytochrome c oxidase subunits I-III (COX1–3) of complex IV. However, adverse alterations in mtDNA copy number or integrity are closely associated with the development of metabolic diseases, such as diabetes (86). Abnormalities in mtDNA copy number have been observed in patients with skeletal muscle atrophy (87) and cardiovascular diseases, which are closely associated with mitochondrial dysfunction (88). Decreased mtDNA copy number downregulates *PGC-1α* and *TFAM* expression and eventually inhibits mitochondrial biogenesis (89). A previous study revealed that castration-induced androgen deficiency decreased the copy number of mtDNA (about 38%) in the skeletal muscle of small pigs (82). Androgens regulate the transcription of mtDNA through AREs, and a study using silico genome analysis (SGA) reported that specific sequence binding sites for AR may be present in mtDNA and that AR can recognize, bind, and even act as transcription factors to regulate mitochondrial gene transcription (84).

Additionally, it is important to note that AR may play detrimental roles in multiple mitochondrial processes through localization to mitochondria in prostate tissues and cell lines. In detail, AR is imported into and localizes to mitochondria with the help of a genuine 36-amino-acid-long mitochondrial localization signal at the N terminus and impedes mtDNA content, TFAM expression, and the expression, assembly, stability, and activity of oxidative phosphorylation (OXPHOS) (90). The mitochondrial stress increases the expression of AR and its translocation to mitochondria, which indicates an intricate relationship between AR and mitochondria.

In summary, androgens/AR signaling regulates mitochondrial biogenesis by enhancing the PGC-1α/NRF-1/TFAM pathway and affecting mtDNA copy number and OXPHOS through localization to mitochondria.

### 4.2 Androgens/AR regulate the mitochondrial quality control system

#### 4.2.1 Androgens and AR affect the mitochondrial morphology

Normal mitochondrial morphology is a key factor in maintaining mitochondrial quality. Mitochondria have an outer membrane (OMM) and an inner membrane (IMM). The IMM folds into cristae toward the mitochondrial matrix and is the carrier of the ETC, which pumps protons across the IMM to form an electrochemical gradient that promotes ATP production. Adverse changes in mitochondrial cristae are closely associated with decreases in ATP production and thermogenesis. Abnormal mitochondrial morphology can inhibit mitochondrial OXPHOS and alter calcium homeostasis and mitochondria-mediated apoptosis, leading to mitochondrial dysfunction and mitochondria-related diseases (91).

Androgens/AR signaling affects mitochondrial morphology. Mitochondrial swelling and a decrease in mitochondrial cristae and mitochondrial length have been reported in cardiomyocytes of rats with myocardial infarction; castration aggravated the above conditions, while exogenous testosterone supplementation effectively alleviated these abnormalities (17). In addition, mitochondrial vacuolization and reduced mitochondrial OXPHOS levels were also found in the myocardium of ARKO mice (92) and rats with obesity (60), further suggesting the vital role of androgens/AR signaling in mitochondrial morphology, given the strong connection between obesity and decreased androgens/AR levels in males. These findings were also verified by clinical studies, in which the abnormal morphology of mitochondria and decreased oxygen consumption levels were observed in the liver of men with hypogonadism, and exogenous testosterone supplementation notably improved these changes (93).

These results suggest a protective effect of testosterone on mitochondrial morphology in males. However, the specific mechanism by which androgens regulate mitochondrial cristae is not yet clear, and the regulation of the expression of genes related to the mitochondrial cristae structure, such as optical atrophy and cardiolipin, may be included, but further studies are needed to confirm this.

#### 4.2.2 Androgens/AR affect the mitochondrial dynamics

Mitochondria are dynamic organelles that can be divided into multiple fragmented mitochondria or fused to form an interconnected mitochondrial network to maintain cellular energy homeostasis in response to changes in the cellular environment. This process, including fission and fusion, is known as mitochondrial dynamics, which reflects the quality of mitochondria (94). Mitochondrial fusion occurs at both the IMM and OMM to fuse multiple healthy mitochondria into a larger mitochondrion. The damaged mitochondria were separated from healthy ones in the fission process. These processes are regulated by guanosine-5’triphosphate (GTP)-binding proteins, including mitofusin1/2 (MFN1, MFN2, regulate OMM fusion), optical atrophy 1 (OPA1, promotes IMM fusion), and dynamin-related protein 1 (DRP1, is involved in mitochondrial fission). Any abnormality in these molecules can cause an imbalance in mitochondrial dynamics (e.g., excessive mitochondrial fusion or fission), and can ultimately lead to disruptions in mitochondrial structure and function (17). The *Mfn2* gene knockout in the mouse brain not only results in mitochondrial swelling, fragmentation, and structural disruption of mitochondrial cristae in hypothalamic and cortical neurons but also causes significant downregulation of ETC complexes (95). The overexpression of MFN2 enhances mitochondrial fusion to prevent mitochondrial fragmentation (96) and alleviates the decrease in mitochondrial membrane potential and excessive ROS induced by angiotensin II (97). OPA1 is involved in the regulation of mitochondrial cristae morphology (98–100). In the presence of nutrient deficiency or decreased cellular ATP levels, OPA1 tightens the arrangement of mitochondrial cristae, promotes the aggregation of ATP synthase, and increases ATP production (101). Reduction of OPA1 expression directly inhibits OXPHOS and mitochondrial function (102).

The imbalance in mitochondrial dynamics caused by abnormal GTP-binding protein levels is closely associated with the development of chronic diseases such as obesity and type 2 diabetes. Impaired mitochondrial fusion and enhanced mitochondrial fission have been reported in mice with *Mfn2* gene knockout, which was accompanied by metabolic disorders such as glucose intolerance, enhanced hepatic gluconeogenesis, and leptin resistance (66, 103). Abnormally elevated levels of DRP1 not only cause mitochondrial fission, mitochondrial fragmentation, and decreased efficiency of mitochondrial ATP production but also impede glucose uptake (104) and glucose-stimulated insulin secretion by inhibiting the translocation of the insulin receptor substrate 1-protein kinase B (IRS1-Akt) pathway and glucose transport proteins in mouse pancreatic β-cells (105). Whereas knockout of the *Drp1* gene in the mouse skeletal muscle enhances mitochondrial oxidative metabolism (106) and insulin signaling (104, 106).

Androgens/AR signaling can affect mitochondrial dynamics by regulating GTP-binding protein levels. In the hippocampus and substantia nigra of male rats, castration-induced androgen deficiency was closely associated with decreased expression levels of mitochondrial MFN2, OPA1, mitochondrial complex I/III activity, and ATP production (107). In addition, testosterone deprivation downregulates the protein levels of MFN2 and OPA1 in obese rats, and mRNA levels of *Opa1* and *Mfn2* in the tibialis anterior muscle of mice, as well as upregulates DRP1 expression levels (17, 108). Similar results were reported in studies using cultured C_2_C_12_ myoblasts, in which testosterone supplementation promoted mitochondrial fusion by upregulating OPA1 protein levels (84).

In summary, androgens and AR can promote mitochondrial fusion and prevent fragmentation by elevating the fusion-related proteins and inhibiting the fission-related proteins, ultimately affecting the quality and function of mitochondria in males.

#### 4.2.3 Effect of androgens/AR on mitophagy

Mitophagy is a process whereby dysfunctional mitochondria are engulfed by autophagosomes and eventually cleared by lysosomal degradation after mitochondrial fission to maintain normal intracellular mitochondrial levels and metabolism. The phosphatase and tensin homolog (PTEN)-induced kinase 1 (PINK1)/E3 ubiquitin ligase Parkin signaling is a major pathway regulating mitophagy in mammals (109, 110). Under stress conditions, PINK1 promotes Parkin recruitment to the OMM to ubiquitinate mitochondrial proteins and polyubiquitin chains are subsequently phosphorylated by PINK1 as an initiation signal for autophagy. The phosphorylated polyubiquitin chains are recognized by mitochondrial autophagy receptors, such as BNIP3 (BCL2/Adenovirus E1B 19 kDa Interacting Protein 3) and p62, and mediate mitochondrial autophagy by binding to lipidated microtubule-associated protein light chain 3 II (LC3 II). The upregulation of *BNIP3* expression promotes mitophagy (111).

Androgens play vital roles in the regulation of mitophagy. The marked increases in protein levels of BNIP3, P62, and PARKIN were clearly observed in the tibialis anterior muscle of mice with castration (112), and the increased ratio of LC3 II/LC3 I in mice with hypogonadism, which indicates a strong association between androgen deficiency and enhancement of mitophagy (113). These results suggest that androgens enhance the mitochondrial mass by promoting mitochondrial fusion and inhibiting mitochondrial fission under physiological conditions, whereas the decrease in mitochondrial mass is closely related to the enhancement of mitophagy associated with testosterone deprivation.

### 4.3 Androgens and AR affect the production of mitochondrial ATP

Mitochondria produce ATP to meet cellular energy needs mainly through OXPHOS, which is carried out in ETC. The ETC consists of five independent mitochondrial complexes, including complexes I-V. Any ETC-related abnormality may have an adverse impact on OXPHOS and the tricarboxylic acid (TCA) cycle, which is a shared process of carbohydrate, fat, and protein metabolism. Mitochondrial complexes I and II in the ETC replenish nicotinamide adenine dinucleotide (NAD^+^) and flavin adenine dinucleotide (FAD), respectively, allowing the oxidative TCA cycle to function (114). Structural abnormalities or dysfunctions of the ETC result in decreased mitochondrial ATP production and abnormal cellular function (115, 116). Decreased activity of complexes I and II is closely associated with reduced ATP production in cardiomyocytes of male obese rats with IR (117), and the inhibition of cytochrome c oxidase and complex IV deteriorates dysfunctional mitochondria and decreases ATP levels (118). This evidence reveals that the intact ETC with normal function is essential for ATP production in mitochondria and for the metabolism of energy substrates.

Androgens/AR signaling can regulate mitochondrial energy production by affecting the ETC. In cultured hepatocytes, flutamide inhibited the activity of mitochondrial complex I and decreased mitochondrial membrane potential (MMP) and ATP production (decreased by about 51.2%) (119, 120). The decrease in MMP can lead to the opening of pores thus increasing mitochondrial membrane permeability (121), and a large amount of H+ pumped into the mitochondrial matrix causes a disruption of proton concentration on both sides of the mitochondrial membrane to inhibit ATP synthesis and accelerate ATP hydrolysis. Evidence from studies *in vivo* suggests that androgen deficiency is closely associated with decreases in ATP synthesis and the activity of complexes I and III in the hippocampus and substantia nigra of castrated male rats (84), which was improved by exogenous testosterone supplementation (16–18). Reduction in ATP and expression levels of mitochondrial complex V and its subunits (e.g., ATP6, ATP8) were found in the substantia nigra of aged rats, which were also reversed by exogenous testosterone (122). Thus, all this evidence reveals the regulatory role of androgens in ATP production, which might be achieved by its influence on ETC. However, how androgens affect ETC has not been clearly demonstrated. We propose that the underlying mechanism behind that impact may be related to the mitigation of cellular oxidative damage and glycolipotoxicity, according to previous evidence from preclinical and clinical studies.

ROS at the physiological level can act as redox messengers involved in the regulation of various cellular activities, such as cell growth, differentiation, proliferation, and apoptosis (123). Mitochondria are the main site of ROS production but are also the target of excessive ROS. The mtDNA is more vulnerable to excessive ROS-related damage to biomolecules, such as DNA repair enzyme proteins, than nuclear DNA (112), which disrupts the ETC and ultimately induces dysregulation of mitochondrial OXPHOS (113). Exposure to high doses of H_2_O_2_ enhances oxidative stress accompanied by decreases in gene levels of mtDNA-encoded mitochondrial respiratory chain-related proteins such as cytochrome c oxidase subunit 1/2 (Cox1/2), NADPH dehydrogenase subunit 1/4 (ND1/4), and CytB (84). Maintaining the oxidative stress balance is vital to the normal function of ETC.

Androgens/AR improve ETC function and maintain mitochondrial ATP production by alleviating oxidative damage. Castration-induced androgen deficiency is strongly associated with the deterioration of ETC in the skeletal muscle, increases in H_2_O_2_ in plasma, and malondialdehyde in cardiomocytes (124), which accelerates cardiomyocyte apoptosis (16, 69). In addition, castration also increased the expression of ROS-production related genes such as Nox1 and GP91PHOX (the catalytic subunit of Nox2) and decreased the levels of antioxidant enzymes such as superoxide dismutase 2 (SOD2), glutathione peroxidase 1 (GPX1), thioredoxin, and peroxiredoxin 5 in the prostate alveolar epithelium of rats (125). Exogenous androgen protects ETC function by alleviating mitochondrial oxidative stress, as evidenced by results showing that testosterone supplementation reduces Nox1 expression, and increases the expression levels of antioxidants (e.g., SOD2, GPX1, thioredoxin, catalase, and glutathione reductase) in the ventral prostate of castrated rats (84, 126). Treatment with AR antagonist flutamide increased H_2_O_2_ levels by approximately 2.4-fold and decreased MMP and ATP production in hepatocytes (120), which further demonstrates the important role of androgens/AR in regulating cellular oxidative stress levels.

Androgens/AR signaling may protect mitochondrial function by alleviating glycolipotoxicity. Glycolipotoxicity has been considered as a potential mechanism linking mitochondrial dysfunction and IR for its detrimental role in insulin secretion from pancreatic β cells and inducing impairment of ETC. High glucose treatment decreased the mitochondrial respiration level in myogenic cells, and glycoliptoxicity in pancreatic β cells reduced the mitochondrial oxygen consumption level (127). Results from a study *in vivo* suggest that continuous low-dose infusion of exogenous glucose and fatty milk induces a glycolipotoxic response in obese rats, causing mitochondrial swelling and cristae rupture in skeletal muscle cells (128). In hypogonadal male or castrated mice, unfavorable changes in pancreatic β-cell function, blood glucose, and blood lipid levels were observed, which were improved by exogenous androgen supplementation. The exogenous testosterone may alleviate glycolipotoxicity-related mitochondrial dysfunction through the regulation of key enzymes involved in glucose and lipid metabolism, including adipose triglyceride lipase (ATGL), phosphoenolpyruvate carboxykinase (PEPCK), and proteins including glucose transport protein 4 (GLUT4) (129).

The regulation of mitochondrial pyruvate carrier (MPC) may also partially explain the mechanism underlying the effects of androgens/AR on mitochondrial function. The MPC is composed of co-stabilizing proteins MPC1 and MPC2 and is located at the IMM. Its main function is to maintain metabolic homeostasis by importing pyruvate into the mitochondrial matrix for utilization in intermediary metabolism in the TCA cycle. This import is a crucial step for the efficient generation of reducing equivalents and ATP, as well as for the synthesis of glucose, fatty acids, and amino acids from pyruvate (130). AR directly regulate the transcription of MPC2 (131). This is supported by the identification of two potential ARE half-sites in the first intron of the MPC2 gene and the confirmation that AR recruitment to these sites is androgen-dependent. The anti-androgen enzalutamide inhibits this recruitment. The regulation of MPC by androgens/AR promotes the direct flow of glycolytic flux into mitochondria, fueling TCA metabolism and subsequently increasing OXPHOS and lipogenesis in PCa cells. Inhibition of MPC decreases AR-driven cellular proliferation, OXPHOS capacity, lipogenesis, and also leads to the swelling of mitochondrial cristae in PCa cells, which is in line with the decreased oxygen consumption and ATP production (131).

**Figure 2:**
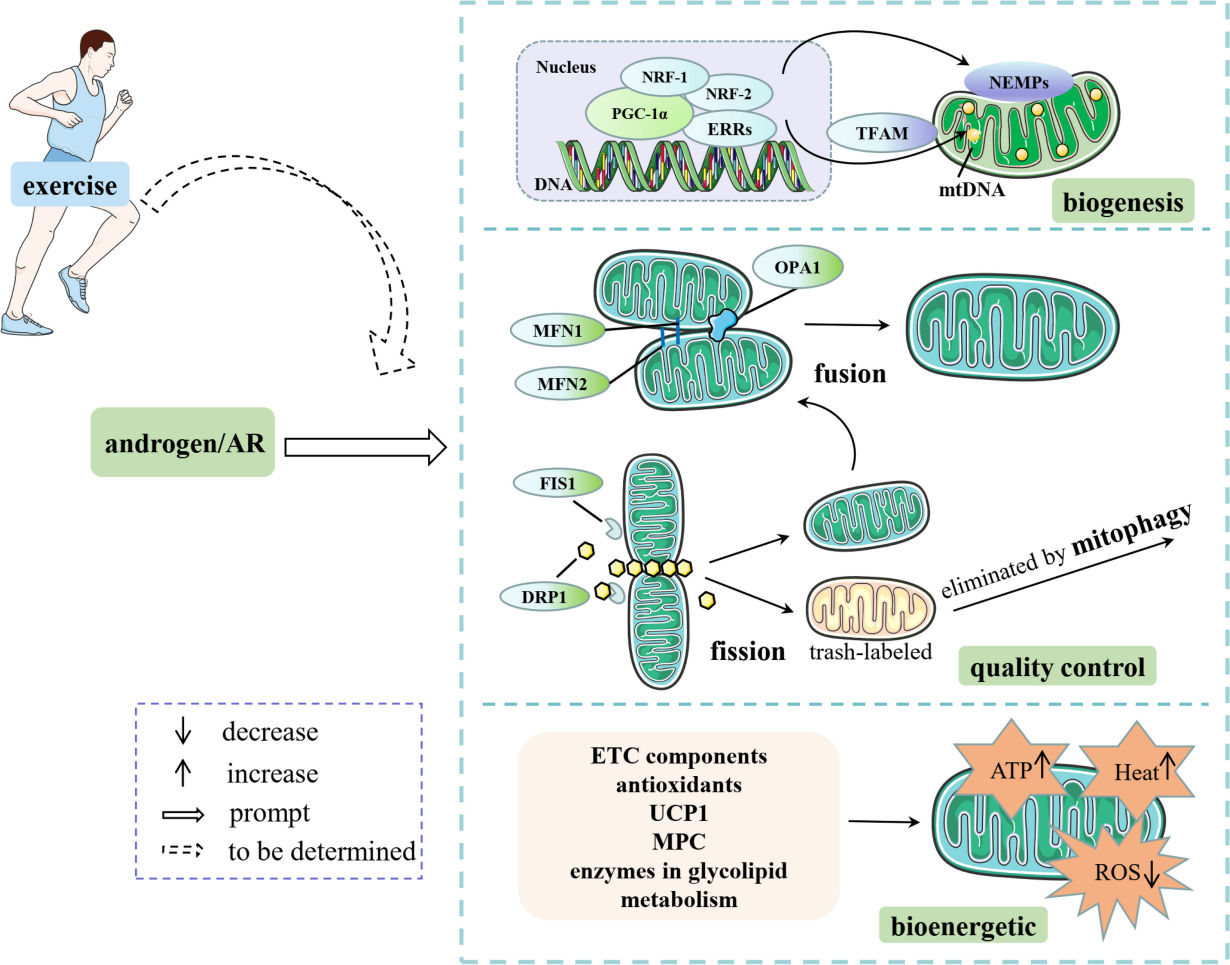
Androgens/AR affect mitochondrial content, quality control and energetic capacity through multiple pathways. Some elements of this figure are from Servier Medical Art, https://smart.servier.com.

In summary, androgens/AR signaling alleviates mitochondrial oxidative stress by regulating the expression levels of mitochondrial respiratory chain-related enzymes such as Cox1/2 and CytB, decreasing the levels of peroxisomes (e.g., H_2_O_2_ and MDA), increasing the antioxidant levels (e.g., SOD2 and GPX1) and enhances OXPHOS capacity through regulating MPC. In addition, androgens promote lipolysis and glucose uptake by upregulating the expression of key enzymes and proteins involved in glucose and lipid metabolism, such as ATGL and GLUT4, to alleviate glycolipotoxicity, thus eventually achieving a protective effect on the structural integrity and function of the electron transfer chain (**Figure 2**).

## 5 Excessive androgens/AR impairs mitochondrial quality control and function in females

Different from the androgen deficiency-related negative impact on mitochondria in males, excessive androgens and/or overactivation of AR play detrimental roles in mitochondrial quality control and functions in females. This is mostly reported in women or female animals with PCOS. Recent studies reported that excessive DHEA or DHT induced decreased mitochondrial number in the ovarian granulosa cells and oocytes of rats with PCOS (132, 133). In obese mice with PCOS, hyperandrogenemia is strongly associated with the disruption of mitochondrial structure in the oocytes (134). In terms of mitochondrial dynamics, excessive androgens are responsible for the decreased mitochondrial fusion and increased fission-induced fragmentation, as evidenced by the marked increase in *Drp1* gene expression in the liver of mice with PCOS (135). Interestingly, the effect of androgens on mitochondrial dynamics may be influenced by the metabolic status and dose of the exogenous androgen, for the failure of testosterone exposure to change these dynamics-related proteins (e.g., DRP1, OPA1, and MFN2) in the skeletal muscle of healthy young women (136).

Additionally, excessive levels of androgens have been found to induce increased mitophagy and oxidative stress in females. This was demonstrated by elevated transcript levels of autophagy-related receptors NIX and Ras Homolog Enriched in Brain (RHEB), as well as autophagy genes such as *Atg9* and *Atg12*, in ovarian granulosa cells of females with PCOS or in mice with PCOS induced by DHT (137, 138). Furthermore, a significant reduction in the protein levels of the antioxidant SOD1 was observed in the livers of female mice with DHT-induced PCOS (135). These detrimental effects of excessive androgens on mitochondria are primarily mediated through AR. This is supported by findings that intracellular ATP content, mtDNA copy number, and protein levels of NRF1 and TFAM were significantly decreased in pancreatic islets of female rats treated with DHT, but these effects were prevented by pre-treatment with the AR antagonist flutamide (139).

Taken together, these studies suggest the sex-dependent effects of androgens/AR on mitochondria but the possible reasons remain largely elusive. A recent review proposed a possible explanation, suggesting that excessive androgen levels may induce mitochondrial overactivation and ATP production surplus in females with PCOS through a synergistic effect with estrogen (140), considering the overlapping activities of androgens and estrogen in regulating mitochondrial function (77). This excessive mitochondrial activity could potentially lead to mitochondrial dysfunction.

## 6 Changes in androgens/AR and mitochondria may partly explain the effects of exercise on glucose and lipid metabolism

Exercise has been widely reported to ameliorate obesity and diabetes by improving glucose and lipid metabolism. A recent review summarized that combined exercise improves glycemic control, weight loss, and insulin sensitivity among patients with type 2 diabetes and concurrent overweight/obesity (141). Pre-clinical study has also revealed that aerobic exercise can improve glucose and lipid metabolism in male rats with obesity or diabetes by decreasing levels of fasting blood glucose, insulin, TG, and LDL cholesterol (142).

Androgens/AR and mitochondria may be involved in the exercise-induced effects on glucose and lipid metabolism, considering the regulatory role of exercise in both androgens/AR and mitochondria. It is important to note that excessive exercise (e.g., overtraining) is widely reported to decrease levels of androgens (143) and AR (144). Appropriate exercise has been found to increase endogenous androgen production (145, 146), promote skeletal muscle hypertrophy (147), and reduce age-related decreases in androgens in aging men (148). The administration of exogenous androgens, such as mesterolone, has been shown to increase the cross-sectional area of oxidative muscle fibers, particularly when combined with endurance exercise (149). Exercise also regulates AR protein levels in the skeletal muscle. Chronic aerobic exercise has been found to increase AR protein levels, which in turn affects glucose and lipid metabolism in several ways. Firstly, the upregulation of AR induced by exercise is associated with improvements in muscle mass, suggesting higher energy expenditure (147, 150). Secondly, exercise-related AR activation has been shown to improve blood glucose and lipid levels in male rats with obesity or diabetes by regulating the protein levels of key enzymes involved in glucose and lipid metabolism, such as PEPCK (151). Additionally, AR may play a role in the impact of exercise on metabolism by regulating other key enzymes, such as GLUT4 (152), lipoprotein lipase (LPL), and ATGL (153), as previous evidence suggests that both exercise and AR can regulate these enzymes (85). Regarding mitochondria, a comprehensive review summarized that exercise enhances physical performance and confers health benefits largely through coordinating improvements in the content, structure, and function of mitochondria (154). The utilization of regular exercise programs including both endurance and resistance training can be an effective strategy for managing sarcopenic obesity through improving mitochondrial function (155).

In summary, these results highlight the important regulatory impacts of exercise on androgens/AR and mitochondria. However, whether androgens/AR are involved in the exercise-induced improvement of mitochondrial function and the subsequent regulation of glucose and lipid metabolism remains elusive at present.

## 7 Conclusions

Mitochondrial dysfunction and IR are closely related to each other and are major factors in the development and progression of metabolic diseases such as obesity and diabetes mellitus. Androgens regulate glucose and lipid metabolism in a sex-dependent manner, and their mechanism is strongly associated with their impact on mitochondria. In this study, we analyzed the role and mechanism of androgens/AR signaling in regulating glucose and lipid metabolism from a mitochondria-centered perspective. In short, androgens/AR promote mitochondrial quantity by activating biogenesis-related signaling and maintain mitochondrial function through a positive effect on the morphology of mitochondria and integrity of the mitochondrial respiratory chain in males. In females, excessive androgen plays a detrimental role in the mitochondrial quality control system. The overactivation induced by a synergistic effect with estrogen may partly explain the detrimental impact of excessive androgen on mitochondria in females but needs further validation.

In addition, it is widely accepted that exercise alleviates or even reverses disorders in glucose and lipid metabolism in individuals with chronic diseases, such as obesity and diabetes, by improving mitochondrial function, which is accompanied by enhanced androgens/AR signaling. However, whether androgens/AR are involved in exercise-induced improvement of mitochondrial function and the subsequent regulation of glucose and lipid metabolism is elusive at present but deserves further investigation in the future.

## Author contributions

LY: Conceptualization, Writing – original draft, Writing – review & editing. SQ: Writing – original draft. ZZ: Conceptualization, Funding acquisition, Supervision, Writing – review & editing.
